# Variability in white blood cell count during uncomplicated malaria and implications for parasite density estimation: a WorldWide Antimalarial Resistance Network individual patient data meta-analysis

**DOI:** 10.1186/s12936-023-04583-6

**Published:** 2023-06-06

**Authors:** WorldWide Antimalarial Resistance Network White Blood Cell Count in Malaria Study Group, Elke Wynberg, Robert J. Commons, Georgina Humphreys, Hazel Ashurst, Rebekah Burrow, George O. Adjei, Martin Adjuik, Nicholas M. Anstey, Anup Anvikar, Kevin J. Baird, Bridget E. Barber, Hubert Barennes, Elisabeth Baudin, David J. Bell, Delia Bethell, Tran Quang Binh, Isabelle Borghini-Fuhrer, Cindy S. Chu, Andre Daher, Umberto D’Alessandro, Debashish Das, Timothy M. E. Davis, Peter J. de Vries, Abdoulaye A. Djimde, Arjen M. Dondorp, Grant Dorsey, Jean-François F. Faucher, Carole Fogg, Oumar Gaye, Matthew Grigg, Christoph Hatz, Piet A. Kager, Marcus Lacerda, Moses Laman, Andreas Mårtensson, Herv Ignace Eby Menan, Wuelton M. Monteiro, Brioni R. Moore, Francois Nosten, Bernhards Ogutu, Lyda Osorio, Louis K. Penali, Dhelio B. Pereira, Awab G. Rahim, Michael Ramharter, Issaka Sagara, Birgit Schramm, Lorenz Seidlein, Andre M. Siqueira, Sodiomon B. Sirima, Peter Starzengruber, Inge Sutanto, Walter R. Taylor, Offianan A. Toure, Jürg Utzinger, Innocent Valea, Giovanni Valentini, Nicholas J. White, Timothy William, Charles J. Woodrow, Caitlin L. Richmond, Philippe J. Guerin, Ric N. Price, Kasia Stepniewska

**Affiliations:** Centre for Tropical Medicine and Global Health, University of Oxford, Oxford, United Kingdom New Richards Building, Old Road Campus, Roosevelt Drive, OX3 7LG

**Keywords:** Malaria, White blood cell, Leukocyte, Parasitaemia, Microscopy

## Abstract

**Background:**

The World Health Organization (WHO) recommends that when peripheral malarial parasitaemia is quantified by thick film microscopy, an actual white blood cell (WBC) count from a concurrently collected blood sample is used in calculations. However, in resource-limited settings an assumed WBC count is often used instead. The aim of this study was to describe the variability in WBC count during acute uncomplicated malaria, and estimate the impact of using an assumed value of WBC on estimates of parasite density and clearance.

**Methods:**

Uncomplicated malaria drug efficacy studies that measured WBC count were selected from the WorldWide Antimalarial Resistance Network data repository for an individual patient data meta-analysis of WBC counts. Regression models with random intercepts for study-site were used to assess WBC count variability at presentation and during follow-up. Inflation factors for parasitaemia density, and clearance estimates were calculated for methods using assumed WBC counts (8000 cells/µL and age-stratified values) using estimates derived from the measured WBC value as reference.

**Results:**

Eighty-four studies enrolling 27,656 patients with clinically uncomplicated malaria were included. Geometric mean WBC counts (× 1000 cells/µL) in age groups < 1, 1–4, 5–14 and ≥ 15 years were 10.5, 8.3, 7.1, 5.7 and 7.5, 7.0, 6.5, 6.0 for individuals with falciparum (n = 24,978) and vivax (n = 2678) malaria, respectively. At presentation, higher WBC counts were seen among patients with higher parasitaemia, severe anaemia and, for individuals with vivax malaria, in regions with shorter regional relapse periodicity. Among falciparum malaria patients, using an assumed WBC count of 8000 cells/µL resulted in parasite density underestimation by a median (IQR) of 26% (4–41%) in infants < 1 year old but an overestimation by 50% (16–91%) in adults aged ≥ 15 years. Use of age-stratified assumed WBC values removed systematic bias but did not improve precision of parasitaemia estimation. Imprecision of parasite clearance estimates was only affected by the within-patient WBC variability over time, and remained < 10% for 79% of patients.

**Conclusions:**

Using an assumed WBC value for parasite density estimation from a thick smear may lead to underdiagnosis of hyperparasitaemia and could adversely affect clinical management; but does not result in clinically consequential inaccuracies in the estimation of the prevalence of prolonged parasite clearance and artemisinin resistance.

**Supplementary Information:**

The online version contains supplementary material available at 10.1186/s12936-023-04583-6.

## Background

In 2021 there were an estimated 247 million cases of malaria worldwide, of which 619,000 had a fatal outcome [1]. *Plasmodium falciparum* infection accounts for the greatest malarial mortality, although *Plasmodium vivax* infection also results in substantial direct and indirect morbidity and associated mortality [2]. The haematological features of acute malaria underpin several key clinical characteristics of the disease. For instance, anaemia and thrombocytopenia are considered hallmark features of acute malarial illness, especially in those with severe disease [3–6]. Reductions in white blood cell (WBC) count have also been reported [7–9]. However, the clinical significance of changes in WBC counts have not been well-described.

Previous studies show that the WBC count can vary tenfold between individuals [7]) and can fluctuate considerably during the course of acute malaria [8–10]. Leukopenia has been reported among children and non-immune adults [11–16], and is thought to be attributable to lymphopenia secondary to redistribution to marginal pools such as the spleen [15]. Leukocytosis can also occur, and is associated with concurrent bacterial infection [7] and severe malaria [11]. Whilst it is known that, independent of infection, children have a higher WBC count than adults [17], other determinants of WBC count during malaria are poorly understood. For example, in Thailand, WBC counts were consistently lower at presentation in those with *P. falciparum* compared to those with *P. vivax* infection [18], yet the opposite was observed in India [9]. The relevance of other factors such as malaria immunity and nutritional status is unclear [18].

Understanding variation in WBC count during acute malaria can have important clinical implications if the WBC is used for the estimation of the parasite density. The World Health Organization (WHO) recommends that peripheral parasitaemia should be quantified by microscopic blood film examination using either thin or thick blood films. Thin films are recommended when quantifying high parasite densities (approximately > 16,000 parasites/µL or >0.3% parasitaemia) [19]. In low to moderate density infections, thick blood film examination in which parasites are counted against WBC is more accurate [20]. In order to calculate the parasite density using a thick blood film, the number of parasites seen per 200 or 500 WBC is counted, expressed per 1 cell and multiplied by the measured total circulating WBC count. However, the ability to measure WBC count is rarely available in remote rural communities where the main burden of malaria exists. Under these circumstances, the WHO recommends using the high-power field (HPF) method [20] instead of the pre-2015 recommendation in which a WBC count of 8000 cells/µL was assumed. The HPF method uses a fixed volume of blood at 1000 × magnification to count parasites. It has desirable properties as it is unbiased with variability decreasing with number of fields examined [21] and has been shown to have a better accuracy compared to WBC methods assuming a count of 8000 cells/µL [21–23]. Whilst the Earle-Perez method, which does not require a known WBC count, has also been shown to produce reliable parasitaemia estimates when using a thick film [24], inter-rater reliability is slightly poorer and in practice this method is rarely used [25].

Despite the 2015 change in guidelines, and the disputed accuracy of assuming a WBC count value of 8,000 cells/µL [26–34], a recent systematic review of microscopy methods used in antimalarial efficacy studies since 2015 reported that a assumed WBC count was used to estimate parasite density in 91% of studies with thick smear microscopy [25]. In these studies, inaccurate estimates of parasitaemia may affect the quantification of parasite clearance and thus early indicators of declining antimalarial efficacy [35]. In addition, although thin smears are more accurate than thick films in the quantification of high parasite counts, 61% of studies included in the above mentioned review [25] used only the WBC method, and it is important to consider the clinical implications of inaccurate estimation of parasitaemia in *P. falciparum* infections. Falciparum hyperparasitaemia is associated with increased mortality [36] and is one of the WHO’s major criteria for diagnosing clinically severe malaria and indication for parenteral treatment [37]. Therefore accurate determination of falciparum parasite count is of great clinical importance.

This individual patient data (IPD) meta-analysis aimed to characterize the WBC count during acute malaria and describe the consequences of these changes on estimates of parasite density and clearance.

## Methods

### Data acquisition

The WorldWide Antimalarial Resistance Network (WWARN) repository contains data from antimalarial efficacy studies for the treatment of uncomplicated malaria. Data were standardized using the methodology described in the WWARN Data Management and Statistical Analysis Plan [38]. Studies included in previous WWARN haematological individual patient data (IPD) meta-analyses [39, 40] were considered for inclusion in this analysis, if WBC count and parasite density in *P. falciparum* and/or *P. vivax* infection had been measured on the day of enrolment (day 0). Individual patient data were only included if the following parameters were available: age at enrolment, sex, and enrolment date. Within studies, pregnant women, non-immune returning travellers with malaria and those with mixed infection were excluded due to small numbers.

Following identification of eligible studies, permission to include data in the current study was granted by an independent Data Access Committee [41] or the study investigators according to each study’s investigators previous selection [42]. Standardized IPD from eligible studies were then collated into a single dataset.

### Definitions

Day of enrolment into the study was defined as day 0. Malaria prevalence rates for *P. falciparum* were obtained for study sites and enrolment year from the Malaria Atlas Project (MAP) [43], updated in 2018. Rates are age-standardized to children aged 2–10 years and resulting estimates, representing transmission intensity, are categorized into low (parasite rate [PfPR_2-10_] ≤ 15%), moderate (PfPR_2-10_ 15- to < 40%) and high (PfPR_2-10_ ≥ 40%) as used in previous WWARN analyses [39]. For *P. vivax* studies, parasite prevalence correlates strongly with the regional *P. vivax* relapse periodicity and geographic region. Thus regional relapse periodicity provides a substitute measure of both geographic and parasite transmission intensity differences. Short relapse periodicity was defined as a median time to patient relapse of 47 days or less [44]. Abnormal WBC counts were approximated to the age-stratified UK National Health Service (NHS) recommendations for adults and children [45, 46]: leukopenia and leukocytosis were defined respectively as having a WBC count of < 6000 and > 18,000 cells/µL for infants < 2 years, < 5000 and > 15,000 cells/µL for children aged 2 to 16, and < 4000 and > 11,000 cells/µL for adults aged 16 and above. Furthermore, Division of AIDS (DAIDS) grading [47] was used to define leukopenia with potential consequences to patient safety following initiation of treatment. For individuals aged 7 days or older, a low WBC count was defined by DAIDS as: mild (2000–2499 cells/µL), moderate (1500–1999 cells/µL), severe (1000–1400 cells/µL) and potentially life-threatening (< 1000 cells/µL). Moderate anaemia was defined as a haemoglobin concentration of < 10 g/dL and severe anaemia as < 7 g/dL. For studies where haematocrit only was measured, the following relationship was used to estimate haemoglobin: haematocrit (%) = 5.62 + 2.60 × haemoglobin (g/dL) [48]. Nutritional status of children aged < 5 years was determined by the weight-for-age indicator using the *igrowup* package [49]. The presence of fever was defined as a recorded core temperature ≥ 37.5°Celsius (°C).

This manuscript refers to asexual parasite counts. Gametocytes (i.e., sexual form parasites) are usually counted using WBC methods, however in the acute malaria infection their densities are much lower and not directly associated with disease severity or patient treatment outcomes. Hyperparasitaemia in *P. falciparum* infection at day 0 was defined as a parasite count of ≥ 100,000/µL in the primary analysis, using the parasitaemia reported in the study. Two additional definitions for hyperparasitaemia were used in the estimation of parasite density analysis: (i) WHO Treatment Guidelines 2015 (≥ 200,000/µL for all regions) and (ii) WHO Treatment Guidelines 2010 (≥ 250,000/µL for high transmission regions; ≥ 100,000/µL for all other regions) [50]. For patients with *P. falciparum* infection, treatment type was stratified into three groups: (i) WHO-recommended ACT regimens for uncomplicated falciparum malaria [51]; (ii) other artemisinin-based regimens including artesunate monotherapy and (iii) non-artemisinin therapies. For patients with *P. vivax* infection, treatment was stratified as follows: (i) WHO-recommended ACT based regimens [51] with or without primaquine; (ii) other artemisinin-based therapies including artesunate monotherapy and those combined with primaquine or chloroquine; (iii) chloroquine monotherapy; (iv) chloroquine-based therapies with either primaquine or doxycycline co-administration; and (v) other drug combinations.

### Analysis of day 0 WBC counts

Analyses of WBC counts were conducted separately for *P. falciparum* and *P. vivax* mono-infections. Forest plots of geometric mean of day 0 WBC count by study site were generated and heterogeneity assessed using *I*^*2*^ statistics, stratified by age group. Study sites with less than 10 participants were excluded. Uni- and multivariable linear regression models with random intercepts for study site were used to assess the association between demographic and baseline clinical parameters and log- transformed day 0 WBC count. The following covariates: age, sex, day 0 parasitaemia, local transmission intensity, presence of anaemia at day 0 and presence of fever at day 0 were considered for inclusion in final models using the strategy recommended by Collet [52]. Briefly, in the first step all variables significant in the univariable analyses were included in the multivariable model, then those not significant in the presence of other variables were removed, and stepwise variable selection was performed to evaluate in turn all variables not included in the model. Likelihood ratio test with p-value < 0.05 was used to compare nested models. Fractional polynomials were used to explore and present the nonlinear relationship between log-transformed day 0 WBC count and continuous covariates (age, day 0 parasitaemia). Residuals from the final model were assessed against fitted values and in quantile-normal plots to assess goodness-of-fit and normality.

### Analysis of changes in WBC count over time

Studies with at least 50% of patients with WBC count measured at day 0 and any of days 2 or 3 (post-treatment), 7, or 14–28 (recovery phase) were included. These inclusion restrictions were undertaken to avoid biased sampling of unwell patients who may have had repeat WBC tests for clinical reasons, and to increase the probability that measurements reflected standard ‘per-protocol’ procedures. Separate univariable mixed effects models, with random intercepts for study site, of the log of WBC ratio between day 0 and any other day were fitted. It was not possible to examine effect of treatment and dose upon WBC count trajectories as the mg-per-kg doses administered were not available for many studies. A multivariable analysis was not conducted as changes in WBC over time were small and not clinically relevant. The proportion of patients who developed DAIDS-defined leukopenia at day 2, 3 and 7 (assuming leukopenia at day 14 and 28 could be confounded by other factors not captured in this dataset) were tabulated by treatment type. Variability in WBC count between study sites was described in terms of coefficient of variation (CV), for log-normally distributed data it is expressed as $\sqrt{\left(\mathrm{exp}\left({\mathrm{s}}^{2} \right)-1\right)}\times 100\%$, where *s* is the standard deviation of log-transformed values of WBC count. CV measures the standard deviation relative to the mean.

### Using WBC to estimate parasite density

The objective of this analysis was to assess the effect of using an assumed fixed value of WBC count on the accuracy of the parasite density and parasite clearance estimation.

Parasite density based on thick smear is calculated using the following formula [20]:

$${\text{Parasite density per }}\mu {\text{L }} = \,{\text{Number of parasites counted }} \times {\text{ total WBC count per }}\mu {\text{L }} \div {\text{ number of WBCs counted}}$$

Hence, the ratio between parasite densities estimated using an assumed WBC count and using the ‘true’ measured WBC count equals the ratio between the assumed WBC count and ‘true’ WBC count, and was described as the “inflation factor”. Three different methods for calculating parasitaemia were compared to the ‘gold standard’ of using the patient’s own measured WBC count: (i) 8000 cells/μL (a value commonly used as the assumed fixed WBC count), (ii) the geometric mean estimated from the multivariable models described above, but omitting day 0 parasitaemia as this was the primary outcome of this analysis, and (iii) the age-stratified (age groups: < 1, 1–4, 5–14 and ≥ 15 years) geometric mean calculated separately for each species. In order to explore the clinical impact of the inflation factor, the proportion of patients identified as being hyperparasitaemic (using three definitions defined above) when using the measured WBC count (deemed the ‘gold standard’) and the three assumed values were compared.

Owing to data limitations, the effect of the method of parasite density calculation on the parasite clearance estimates could only be evaluated using data on days 0 and 2. In this scenario, the slope of parasite decline can be estimated by ${-}\left( {log\left( {P_{2} } \right) {-} log\left( {P_{0} } \right)} \right)/ 48$ where *P*_*i*_ denotes parasitaemia density on day* i*. The inflation factor for the slope estimated using an assumed value of WBC count is additive, does not depend on the assumed value provided the same value is used for all timepoints, and is equal to the ratio of measured WBC count on day 2 and day 0 (for calculation details see Appendix). The distribution of the inflation factor and its effect on the parasite half-life PC_1/2_ [53] defined as $\frac{log\left(2\right)}{slope}$, as well as on the classification of the artemisinin resistance status [54] was evaluated.

All analyses were performed using Stata Statistical Software (StataCorp LCC: Release 17, College Station, TX, USA).

## Results

**Fig. 1:**
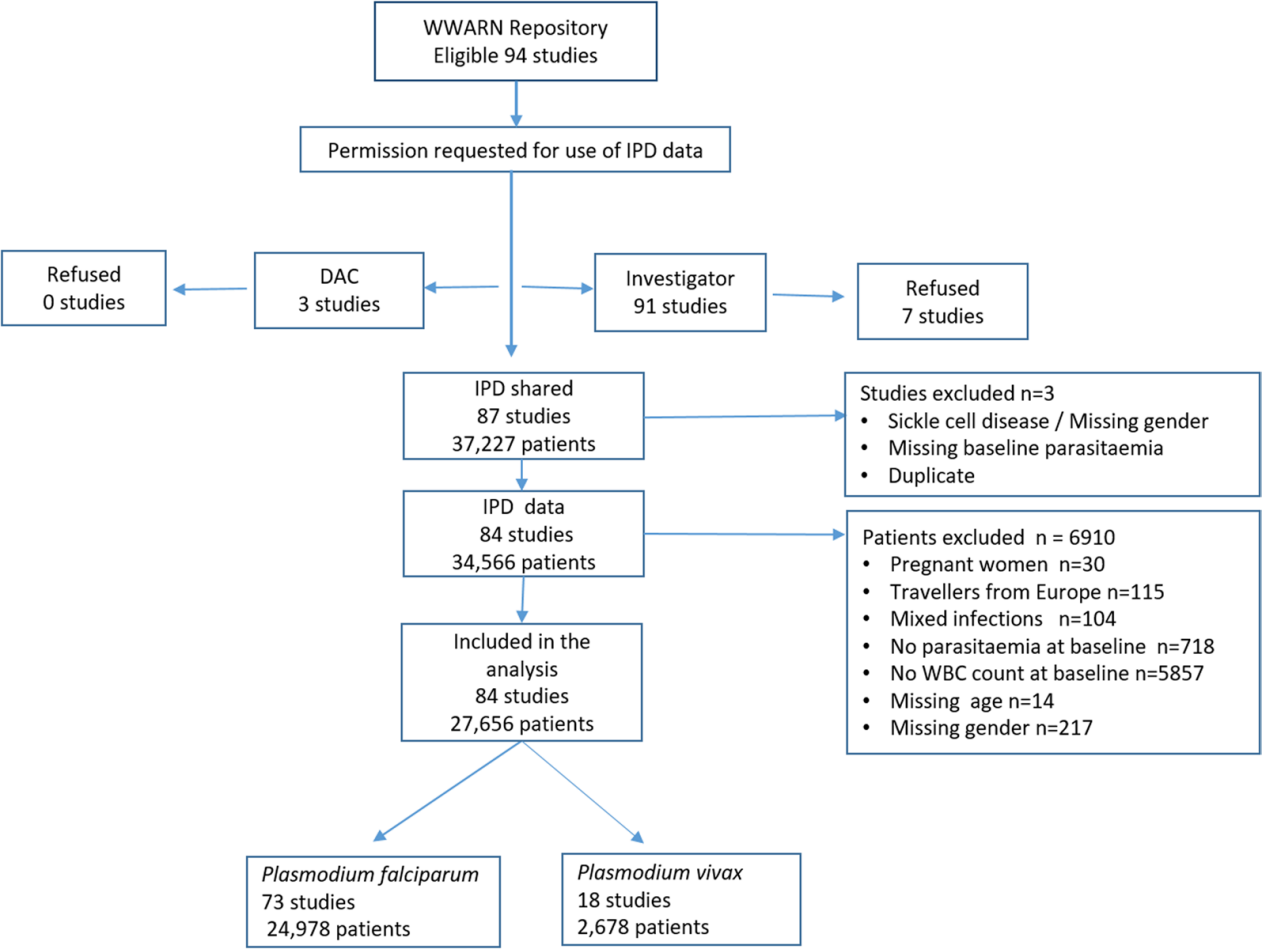
Study profile. Seven of the 84 studies in the analysis include both vivax and falciparum patients

Ninety-three studies met the inclusion criteria, with investigators or the WWARN data access committee agreeing to share data from a total of 87 (92.6%) of these studies (Fig. 1). Two studies were excluded due to missing essential data or meta-data, one was a duplicate. Of the 84 studies remaining, 6661 (19.3%) patients were excluded because of missing age, sex, day 0 WBC count or parasitaemia and 104 were excluded because they were mixed infections. In total 27,656 patients from 30 countries were included in the analysis. Patients were enrolled between 1990 and 2015, at 140 different study sites across Africa (37 studies; 16,747 individuals), the Asia–Pacific region (42 studies; 10,181 individuals) and the Americas (5 studies from Brazil and Colombia; 728 individuals). Details of the included studies and their methodology are presented in Additional file 1: Tables S1–S3. The vast majority of studies used only a thick smear to estimate parasitaemia.

Overall, 24,978 (90.3%) presented with *P. falciparum* mono-infection and 2678 (9.7%) with *P. vivax* mono- infection, their baseline characteristics are summarized in Table 1.

**Table 1:** Patient characteristics at enrolment into studies, by species

Parameter	N	*Plasmodium falciparum*	N	*Plasmodium vivax*
		Median [min; max] or n [%]		Median [min; max] or n [%]
Age (years)	24978	4.9 [0.1; 86.7]	2678	24.6 [0.3; 79.0]
Age group	24978		2678	
< 1 year		1044 [4.2]		2 [0.1]
1–4 years		11606 [46.5]		168 [6.3]
5–14 years		5797 [23.2]		501 [18.7]
15 + years		6531 [26.1]		2007 [74.9]
Male sex	24978	14061 [56.3]	2678	1875 [70.0]
Underweight^1^	12549	2280 [18.2]	170	49 [28.8]
WAZ score^1^	12549	− 1.0 [− 5.8; 4.9]	170	− 1.2 [− 4.6; 2.0]
Wasted^2^	7943	792 [10.0]	34	5 [14.7]
WHZ score^2^	7943	− 0.32 [− 4.9; 5.0]	34	− 0.8 [− 4.2;2.0]
Temperature (°C)	22875	38.0 [34.0; 42.0]	2060	37.4 [34.5; 42.0]
Fever present^3^	23684	15981 [67.5]	2076	1032 [49.7]
Haemoglobin (g/dL)	18687	10.0 [3.1; 26.0]	2596	12.3 [3.9; 18.9]
Haematocrit (%)	13721	34.0 [8.6; 63.0]	1404	39.2 [19.0; 52.3]
Anaemia^4^	24272		2605	
No anaemia		13695 [56.4]		2292 [88.0]
Moderate anaemia		9196[37.9]		294 [11.3]
Severe anaemia		1381 [5.7]		19 [0.7]
Parasitaemia (/µL)	24978	17072 [7; 1528753]	2678	3002 [16; 77035]
Hyperparasitaemia^5^	24978	3124 [12.5]	N/A	N/A
Transmission intensity [*P. falciparum only*]^6^	24978			
Low		15544 [62.2]		
Moderate		3588 [14.4]		
High		5846 [23.4]		
Relapse periodicity [*P*.*vivax* only]^7^			2678	
Long periodicity				1179 [44.0]
Short periodicity				1499 [56.0]
Region	24978		2678	
Africa		16747 [67.0]		0 [0]
Asia–Pacific		8102 [32.4]		2079 [77.6]
Americas		129 [0.5]		599 [22.4]

^1^ Evaluated only in children < 5 years of age. WAZ = weight of age z-score. Underweight defined as a WAZ < − 2

^2^ Evaluated only in children < 5 years of age. WHZ = weight for height z-score. Wasted defined as WHZ < − 2

^3^ Defined as a day 0 temperature of ≥ 37.5 °C

^4^ Severe anaemia = Hb < 7 g/dL; moderate anaemia = Hb 7 to < 10 g/dL

^5^ *P. falciparum* hyperparasitaemia defined as a parasite count ≥ 100,000/µL as reported in the study

^6^ *P. falciparum* transmission intensity defined according to age-standardised parasite rate, where: Low = 0 to 15%, Medium 15 to 40%, High ≥ 40%

^7^*P.* vivax relapse periodicity defined as: Low ≤ 47 days, High > 47 days

*N* number evaluated. *n*  number with the characteristic. *N/A* not applicable. *WBC* white blood cell

### Variability and determinants of day 0 WBC count

The WBC count at day 0 was log-normally distributed, with a minimum value of 200 cells/µL (a 30-year old female patient from Indonesia with falciparum malaria) and maximum value of 87,000 cells/µL (a 16-year old male from Vietnam with falciparum malaria, which remained clinically uncomplicated over 14 days of available follow-up). WBC count varied considerably by age for both species (Table 2). After stratifying by age group there was significant heterogeneity in WBC counts (Table 2), with all *I*^*2*^ among falciparum study sites > 80% and all *I*^*2*^ among vivax study sites > 75% (Additional file 2: Figs. S1–S7). There was no obvious pattern in WBC values through further stratification by continent or between countries.

**Table 2:** Summary of day0 WBC count in patients with malaria, by *Plasmodium* species

Parameter	*Plasmodium falciparum*		*Plasmodium vivax*	
	N	Geometric Mean [min; max] or n [%]	N	Geometric Mean [min; max] or n [%]
WBC count (/μL)				
All age groups	24978	7300 [200; 87000]	2678	6200 [1200; 29400]
< 1 year	1044	10500 [1800; 48580]	2	7500 [7100; 8000]
1–4 years	11606	8300 [1000; 85000]	168	7000[2900; 22400]
5–14 years	5797	7100 [900; 78000]	501	6500[2000; 25700]
15 + years	6531	5700 [200; 87000]	2007	6000 [1200; 29400]
Leukocytosis	24978	1100 [4.4]	2678	41 [1.5]
Leukopenia	24978	3607 [14.4]	2678	277 [10.3]
Clinical Leukopenia (DAIDS grading)	24978	251 [1.0]	2678	27 [1.0]
Mild		153 [0.6]		23 [0.9]
Moderate		54 [0.2]		2 [0.1]
Severe		40 [0.2]		2 [0.1]
Life threatening		4 [0.02]		0 [0.0]

The geometric mean day 0 WBC count in patients with *P. falciparum* was higher in Africa than in Asia and the Americas (Additional file 1: Table S4), but after adjusting for age this difference was no longer significant. However, lower WBC levels were observed in Africa compared to Asia in adults > 15 years of age (by 9.4% [95%CI 1.3–18]) but not in any younger age groups. Underweight children with falciparum malaria (2280/12,549; 18%) presented with 3.3% (95%CI 1.4–5.1) higher day 0 WBC count compared to well-nourished children (10,269/12,549; 82%) (p = 0.001).

**Fig. 2:**
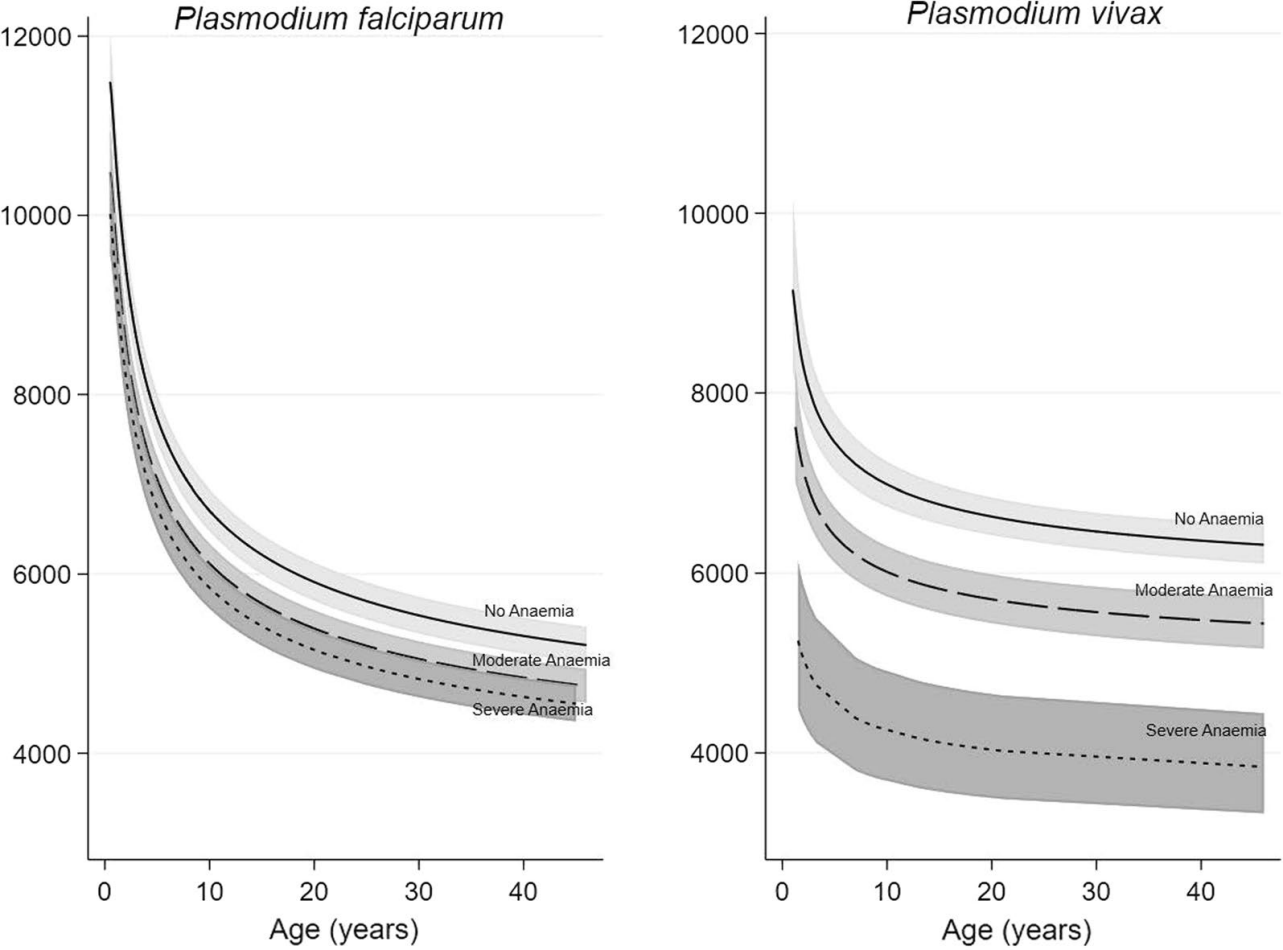
Relationship between day 0 WBC count and age, for different anaemia levels, adjusted for parasite species and relapse periodicity, estimated from the multivariable fractional polynomial model

In multivariable regression analyses the main determinants of WBC count for both species were: age, day 0 parasitaemia and anaemia at enrolment, as well as regional relapse periodicity for patients with *P. vivax* mono-infection and fever in patients with falciparum malaria (Table 3). After adjusting for independent predictors, the geometric mean of the day 0 WBC count decreased with age (Fig. 2). The geometric mean day 0 WBC count was lower in adults compared to children aged 1–4 years of age by 32% (95%CI 30–33) in falciparum malaria and by 17% (95%CI 13–22) in vivax malaria (Table 3). *Plasmodium vivax* patients with severe anaemia had a 36% (95%CI 27–46) lower WBC count compared to those with normal haemoglobin concentrations, while in areas with short relapse periodicity patients presented with a 16% (95%CI 7–26) higher WBC count compared to patients from areas with long relapse periodicity. WBC count was correlated positively with day 0 parasitaemia (with a linear relationship after log-transformation of the two variables), with a 1.9% (95%CI 1.1–2.6) and 5.7% (95%CI 3.5–8.0) increase in WBC count for each tenfold increase in parasite density, for falciparum and vivax malaria, respectively. All other covariates examined were associated with a ≤ 10% difference in day 0 WBC count between groups.

**Table 3:** Multivariable analysis of determinants of day 0 WBC count, by *Plasmodium* species

	*Plasmodium falciparum* n = 23132	*Plasmodium vivax* n = 2605
	% change in day 0 WBC count^1^ (95% CI)	% change in day 0 WBC count^1^ (95% CI)
Age group		
< 1 year	21.7 (18.6, 24.7)	N/A
1–4 years	Reference	Reference^2^
5–14 years	− 16.5 (− 18.1, − 14.8)	− 11.9 (− 16.9, − 6.9)
15 + years	− 31.7 (− 33.2, − 30.1)	− 17.4 (− 22.0, − 12.8)
Log _10_ Parasitaemia (/µL)	1.9 (1.1, 2.6)	5.7 (3.5, 8.0)
Anaemia		
None	Reference	Reference
Moderate	− 6.3 (− 7.4, − 5.2)	− 12.0 (− 15.8, − 8.3)
Severe	− 8.5 (− 10.8, − 6.5)	− 36.4 (− 45.6, − 27.2)
Fever		
No	Reference	N/S
Yes	4.1 (2.9, 5.4)	N/S
Relapse periodicity^3^		
Long	N/A	Reference
Short	N/A	16.4 (6.9, 26.0)

Comparisons with the reference group were significant at p < 0.001 for all variables

^1^% change in day 0 geometric mean of WBC count compared to the reference group

^2^ Includes 2 individuals < 1 year of age

^3^ *P. vivax* relapse periodicity, defined as: Short ≤ 47 days, Long > 47 days

*N/A* Not applicable. *N/S* Not significant. *WBC* white blood cell

The overall CV for WBC count across study sites was 48% in falciparum malaria and 34% in vivax malaria and decreased to 38% and 30% respectively after adjusting for the main determinants and study site. The proportion of total WBC variability explained by study site was higher for falciparum malaria (17% for log-transformed data) than for vivax malaria (7.2% for log-transformed data). No pattern in WBC variability was observed between different age groups or transmission intensities.

### Changes in WBC count during malaria infection

In fifty-one studies (14,138 individuals: 11,958 *P. falciparum*; 2180 *P. vivax*) WBC count was measured for > 50% of participants on day 0 and again on at least day 2, 3, 7, or 14–28; these data were included in the analysis of temporal trends. For both species, the mean changes in WBC count initially fell on day 2–3 before rising (Additional file 1: Table S5). For patients with *P. falciparum*, average changes in WBC count from day 0 did not exceed 11% (Additional file 1: Table S5) at any timepoint. They varied significantly with age group and anaemic status but always remained within 15% in each of subgroups. For patients with *P. vivax* the changes varied with treatment and transmission intensity and remained within 20% of the WBC count on day 0.

Levels and changes over time for different types of WBCs (monocytes, neutrophils and lymphocytes) are presented in Additional file 1: Table S6. Neutrophils peaked at day 0, and fell to at nadir at day 2–3, recovering by day 14–28. In contrast, lymphocytes had a nadir at day 0 but recovered to a steady level by day 2–3. These patterns were observed in both species and for all age groups. No obvious pattern was noted for monocytes.

### Using WBC count to estimate parasite density

**Fig. 3:**
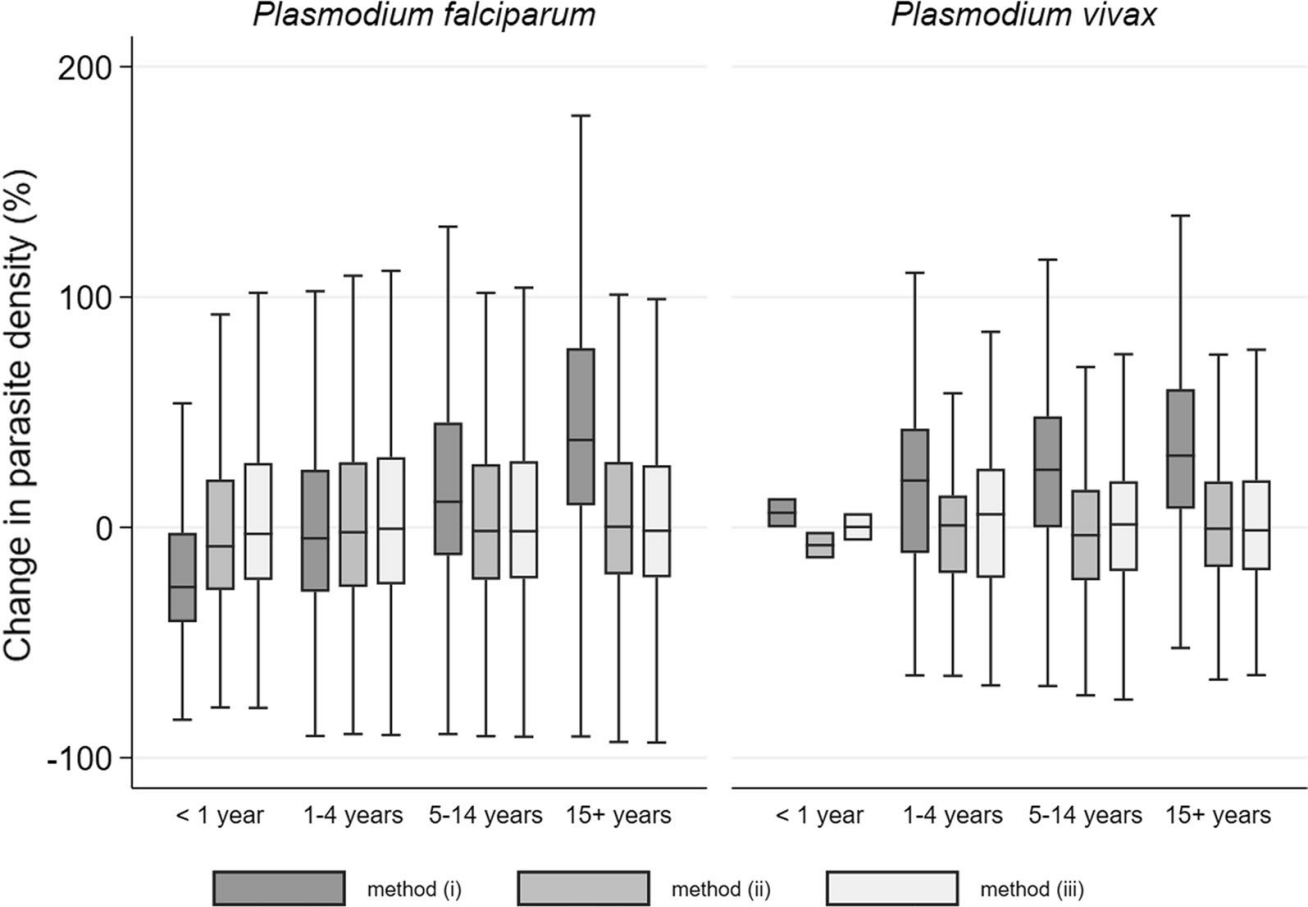
Percentage change (inflation factor) in estimated parasite density in uncomplicated *P. falciparum* and P. vivax* infection, when an assumed WBC count is used compared to using the measured WBC count Method (i) assumes WBC count of 8000 cells/µL; method (ii) assumes WBC count equal to the geometric mean estimated from multivariable model; method (iii) assumes WBC equal to the geometric mean calculated within each age group

When using an assumed WBC count of 8000 cells/µL in the calculation of *P. falciparum* parasite density (method (i), as outlined in Methods), parasite density was underestimated by a median (IQR) of 26% (4–41%) in infants aged < 1 year (n = 969) but overestimated in adults aged ≥ 15 (n = 1707) by 50% (16–91%) (Fig. 3, Additional file 1: Table S7). The bias was smallest and more symmetrical in children 1–5 years of age (n = 10,170) with median (IQR) = − 4.8% (− 28, 25%). Conversely, in patients infected with *P. vivax,* parasite density was overestimated in all age groups: by a median (IQR) of 38% (18–48%) in children aged 1–4 years (n = 70), 25% (− 2.0–48%) in children aged 5–14 years (n = 277), and 33% (11–60%) in adults aged 15 + years (n = 801).

When calculating parasite density using adjusted (method (ii)) and age-stratified (method (iii)) geometric mean WBC counts, the absolute median inflation was < 10% with symmetrical interquartile ranges across all age groups, for both species. However, the variability in the inflation factor of parasite density remained high (Additional file 2: Table S7, Fig. 3).

**Fig. 4:**
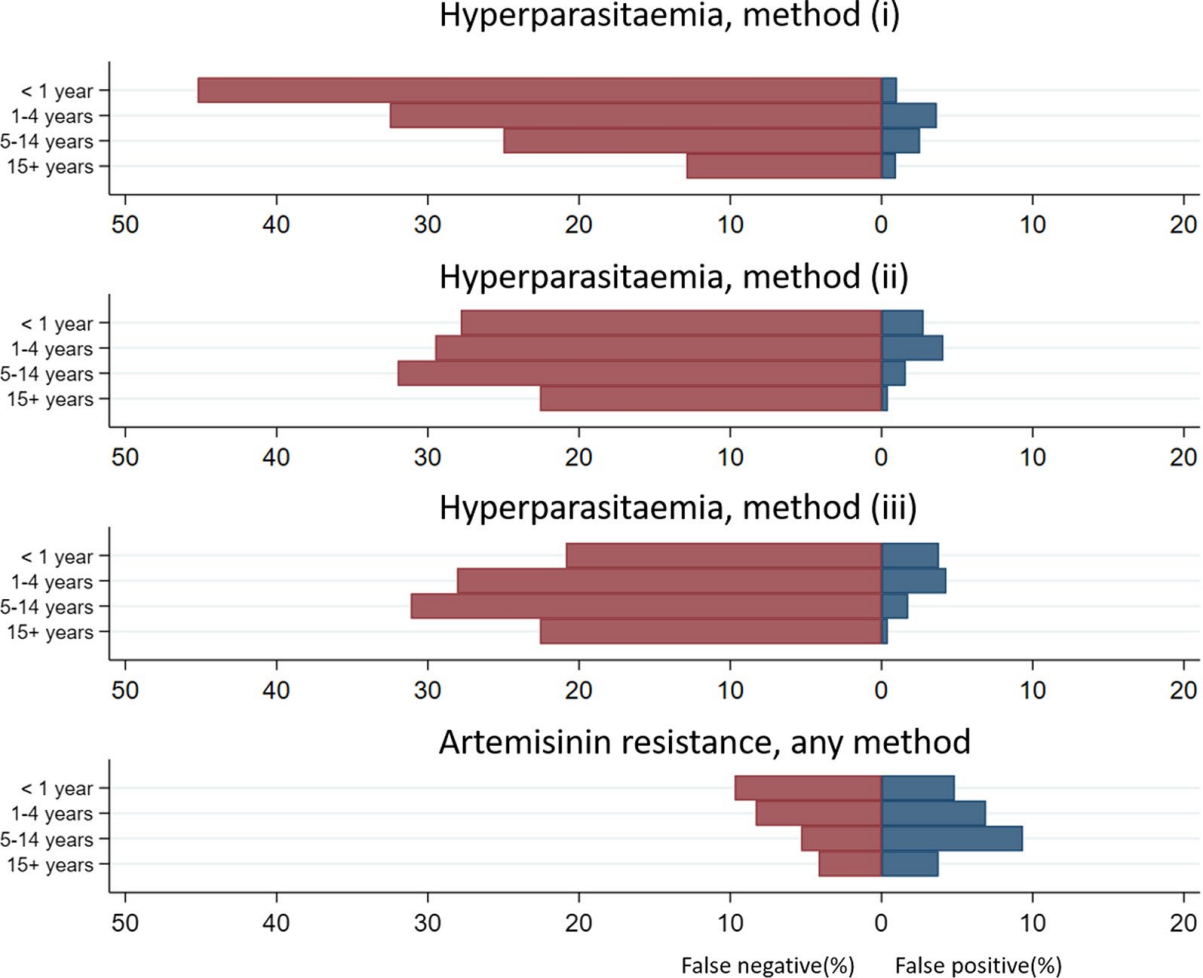
Accuracy in classification of the hyperparasitaemic status (≥ 100,000 parasites/µL) and the artemisinin resistance status in patients with falciparum malaria. Red bars show a proportion (%) of false negatives and blue bars show a proportion (%) of false positives. Method (i) assumes WBC count of 8000 cells/µL; method (ii) assumes WBC count equal to the geometric mean estimated from multivariable model; method (iii) assumes WBC equal to the geometric mean calculated within each age group. For detection of delayed parasite clearance, false negatives were observed only for true parasite clearance (PC_1/2_) of 5 h and false positives for true PC_1/2_ between 6 and 8 h

**Fig. 5:**
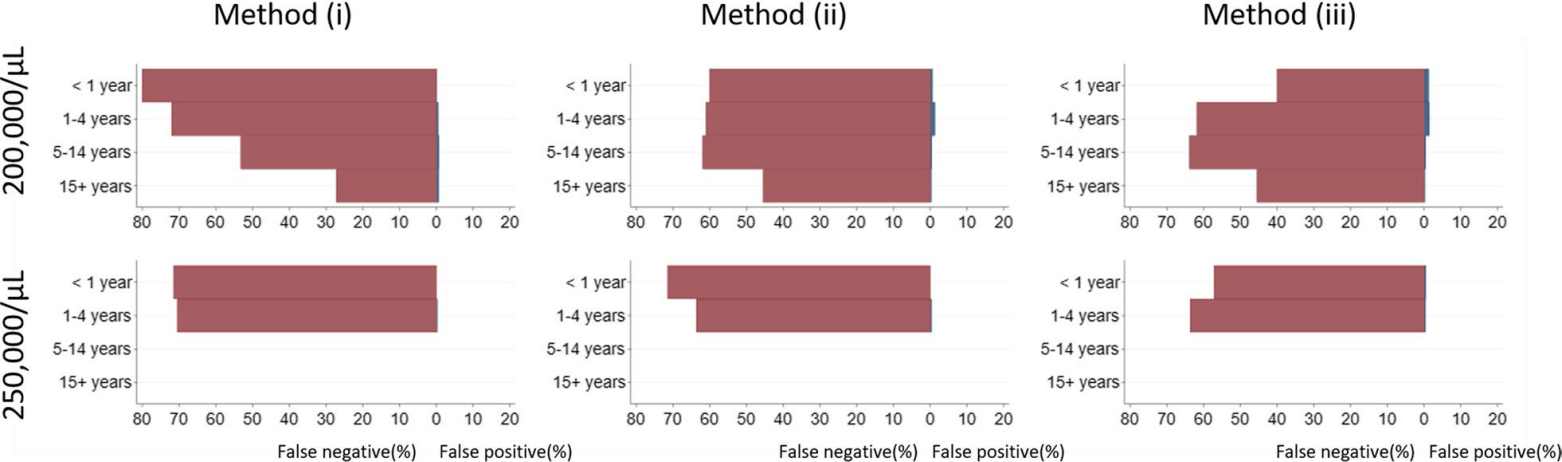
Accuracy in classification of the hyperparasitaemic status in patients with falciparum malaria, using WHO classification. Upper panels show results for 200,000 parasite/µL and lower panels show results for 250,000 parasite/µL cut-offs. Red bars show a proportion (%) of false negatives and blue bars show a proportion (%) of false positives. Method (i) assumes WBC count of 8000 cells/µL; method (ii) assumes WBC count equal to the geometric mean estimated from multivariable model; method (iii) assumes WBC equal to the geometric mean calculated within each age group

Parasitaemia could be re-calculated from counts per slide in 30 studies which used thick films only for parasitaemia estimation in 13,898 patients with *P. falciparum* mono-infection. Overall, of the participants who would be classified as hyperparasitaemic (> 100,000 parasites/µL) using the measured WBC count, 32% (603/1874) would have fallen below the threshold for hyperparasitaemia (false-negative result) by using a WBC of 8000 cells/µL (method (i)), 23% (543/1839) by using an adjusted WBC (method (ii)), and 28% (522/1874) when overall geometric mean of WBC count per age category was used (method (iii)) (Fig. 4, Additional file 1: Table S8). Even higher rates of false negatives were observed when cut-offs for hyperparasitaemia recommended by WHO were used: 200,000 parasites/µL in any region [46] or 250,000 parasites/µL in high transmission areas [47] (Fig. 5). Overall corresponding false negative rates were 70% (318/436) and 71% (36/51) for method (i); 61% (276/429) and 65% (33/51) for method (ii), 60% (261/436) and 63% (32/51) for age adjusted geometric mean method; with negligible false positive rates. For ‘truly’ non-hyperparasitaemic patients, incorrect classification of patients (false-positive result) was below 2% for three methods, overall and in each age group.

The additive inflation factor for the slope of parasite clearance associated with assumed WBC count was small for both species (Additional file 1: Table S9). Corresponding imprecision in PC_1/2_ estimates was positively correlated with PC_1/2_ (Additional file 1: Table S10). PC_1/2_ was underestimated in 46% (1653/3606) of falciparum and 42% (642/1533) of vivax patients. For 75% (2711/3606) of individuals with falciparum malaria and 87% (1328/1533) of individuals with vivax malaria, the difference between the true value and the estimate was within 10% for any PC_1/2_ between 2 and 10 h. A difference of > 20% was observed only for PC_1/2_ between 4 and 10 h in 12% (417/3574) patients with *P. falciparum* and 4.0% (61/1533) *P. vivax* infection. Among those, only 16% (65/417) and 12% (7/61) were underestimated, respectively.

Among falciparum patients with ‘true’ PC_1/2_ equal to 5, 6, 7, or 8 h, 7.8%, 7.4%, 0.1% and 0.03% respectively would have their resistance status misclassified when a 5.5 h cut-off for PC_1/2_ was used to denote artemisinin resistance [55] (Fig. 4). No misclassification resulted for other values of true PC_1/2_.

### Assessment of risk of bias

There was heterogeneity in measurement of WBC count between different studies (Additional file 1: Table S1). Methodological factors potentially contributing to bias are presented in Additional file 1: Table S2. Although many studies were unblinded, WBC count measurement was automated thus minimizing the risk of observer bias. Publication bias was unlikely since WBC measurements were not a primary outcome in any of the publications and WBC counts are unlikely to have influenced the decision to publish. The median (range) proportion of patients for whom WBC was measured at day 0 was 96% (91–100) across studies. Out of 84 studies, 50 were included in the analysis of trends over time, with 26 (52%) of them measuring WBC only on one other occasion until day 14, 22 (44%) on two or three occasions and 2 (4.0%) studies at all time points. The median (range) proportion of patients with WBC measurements at other scheduled time points (included in the analysis) was 94% (54–100) across studies.

## Discussion

To our knowledge, this is the first IPD meta-analysis to characterize WBC count levels in both *P. falciparum* and *P. vivax* malaria across a range of endemic settings. This study shows that for infections caused by either species, age was the most important determinant of day 0 WBC count, but it was also influenced by day 0 parasitaemia, anaemia and, for vivax malaria, regional relapse periodicity. This analysis supports previous findings [27, 28, 32] that using an assumed value of 8000 cells/µL underestimates parasitaemia in children under 5 years (markedly in infants) infected with *P. falciparum* but overestimates parasitaemia in children ≥ 5 years of age and adults, and for all age groups with vivax malaria. This could have substantial consequences in both a clinical and research setting. For instance, in studies using WBC method only to quantify parasite density, more than 60% of patients with parasitaemia > 200,000 parasites/µL were not identified as being hyperparasitaemic when a WBC count of 8000 cells/µL was assumed. This demonstrates that using thick smears with an assumed WBC count, especially in patients with high parasite counts, is a suboptimal way to quantify parasitaemia.

The determinants of day 0 WBC count identified in this study shed light on the haematological response in acute malaria. For example, leukocytosis has been associated with severe malaria and patients with concurrent bacterial infection [56]. The higher day 0 WBC counts observed in hyperparasitaemic patients may, therefore, reflect an immune response to high parasite loads, but is also potentially confounded by comorbid bacterial infection which is estimated to occur in approximately 6% of children with acute malaria [57]. Indeed, analysis of differential WBC counts shows variability in WBC is driven predominantly by neutrophil count. An initial peak in circulating neutrophils has been described previously, particularly among children [16], and may be a marker of concurrent bacterial infection, the most common cause of reactive neutrophilia [58]. However, in the absence of further haematological details regarding this observed neutrophilia, this conclusion remains speculative. In addition, patients with severe anaemia (Hb < 7 g/dL) were observed to have on average a 10% lower WBC count than those with a normal haemoglobin level. Analysis of the bone marrow of individuals infected with malaria has demonstrated bone marrow dysfunction (affecting both erythro- and leucopoiesis) during acute malaria [6], and this biological process may explain the association between anaemia and lower WBC counts.

In patients with *P. vivax* malaria, regional relapse periodicity was another important independent determinant of day 0 WBC count. Patients living in regions with high vivax relapse periodicity may initiate a more rapid immune response to vivax malaria due to immune memory from previous infection, resulting in a higher leucocyte count upon recruitment into the study [59, 60]. However, this association may be confounded by other population characteristics of patients living in these areas which are generally located in South-East Asia and the Western Pacific. Anaemia had a much greater effect on day 0 WBC count in patients with vivax malaria compared to those with falciparum malaria, with a 36.4% lower WBC count observed in vivax patients with severe anaemia (Hb < 7 g/dL) as compared to those with a normal haemoglobin (Hb ≥ 10 g/dL). The spleen and, to a lesser extent, the bone marrow are important reservoirs for the proliferation of vivax parasites [61, 62], which may explain the strong association of vivax patients with lower circulating haemoglobin and WBC counts. Further clinical research is needed explore this clinical relevance of the reservoirs process and their implications on disease progression.

Whilst these descriptive analyses of WBC count are informative for the empirical understanding of malaria, it is important to also highlight the clinical and research implications of using an assumed WBC count in the estimation of parasite density. Firstly, using an assumed count of 8,000 cells/µL resulted in an underestimation of parasitaemia in young children. In a clinical setting, this may have consequences for patient management. For instance, whilst the diagnosis of severe malaria is also based on other clinical criteria, in a child with only minor symptoms, a high parasite load may be an early indication of a poor prognosis and need for parenteral treatment. In a research setting, underestimation of parasitaemia in children may generate a bias towards adverse outcomes among children compared to adults resulting from an undetected high parasite load. Indeed, even though a thin smear should be the method of choice for quantification of high parasite loads, thick smears continue to be commonly-used in research settings [25], owing also to the frequently-applied exclusion criteria of individuals with hyperparasitaemia. In adults, using an assumed WBC count resulted in the overestimation of the proportion of individuals with hyperparasitaemia. In a clinical setting, this may result in overuse of parenteral drugs, wasting a valuable resource. In a research context, moreover, studies often truncate the study population based on parasitaemia, excluding patients with a parasite density exceeding 100,000/μL (in low- and moderate-transmission regions) or 200,000 parasites/μL (in high-transmission regions) [63] due to the increased risk of treatment failure. Should parasitaemia be underestimated, therefore, those with a high parasite biomass may be erroneously enrolled in treatment efficacy studies, thus leading to an overestimation of treatment failure. Wherever possible, studies should aim to quantify parasitaemia at enrolment using the measured WBC count when using a thick blood film, and use a thin smear in patients with a high parasite count, according to WHO guidelines.

The estimation of parasitaemia also has an important role in quantifying the parasite clearance half-life (PC_1/2_), a measure of drug efficacy [64, 65] and marker of artemisinin resistance [54]. Reassuringly, this analysis of parasite clearance half-life found that the absolute imprecision was below 10% for any PC_1/2_ between 2 and 10 h in 75% of falciparum infections. Thus, assuming a WBC count of 8000 cells/μL (or any other assumed value) did not result in clinically consequential inaccuracies in estimation of the prevalence of prolonged parasite clearance and artemisinin resistance.

This study has several strengths and some limitations. It is the first IPD meta-analysis to explore variability in WBC count in patients from all endemic regions and to quantify its effect upon clinically-relevant outcomes. However, the studied population is not representative of all malaria patients, as all studies excluded patients with health conditions that may have caused hospitalization during follow-up, which in turn may also affect WBC count. Other limitations concern studies with different follow up and examination schedules and thus WBC count measured at different timepoints. For instance, only fifty studies measured WBC count after day 0 and half of these only measured it on one other occasion. The WBC data available in the first 24 h after treatment were limited and therefore analysis of the impact of using an assumed WBC count on PC_1/2_ was based on just two measurements, on day 0 and day 2–3. In addition, despite accounting for age and other independent predictors, the variability between patients and between study sites was large. This may be partially explained by the fact that 20–50% of individuals of African descent have been shown to have benign ethnic neutropenia [66]. Moreover, the method of WBC count quantification (using either an automated counter or a manual method) may have led to further variability in WBC count measurements. Finally, many studies used only thick blood smears in this meta-analysis, a finding that was corroborated in a recent review [25]. However, thin blood smears are recommended for quantification of high parasite densities [19, 20]. Therefore, in settings following current guidelines, the proportions of children misclassified as non-hyperparasitaemic may be overestimatedin this study.

## Conclusions

High variability in WBC count between individuals with acute malaria highlights the importance of using measured WBC count to estimate parasitaemia from a thick blood smear whenever possible, in both clinical and research contexts. The use of an assumed value of WBC count resulted in a considerable underestimation of parasite count in children. This could lead to sub-optimal treatment of children with clinically uncomplicated but hyperparasitaemic malaria if a thick rather than thin smear is used to estimate parasitaemia, such as is common in drug efficacy trials. Reassuringly, however, assuming a WBC count of 8000 cells/µL did not result in clinically consequential inaccuracies in the estimation of the prevalence of prolonged parasite clearance and artemisinin resistance. Standardization and quality control of blood film microscopy methods is critical for both optimizing patient management and anti-malarial clinical trials, but is hampered by disparate clinical and laboratory resources. It is, therefore, critical that researchers fully report on microscopy methods to allow for greater transparency between studies, even if standardization is not possible.

### Supplementary Information

**Additional file 1: Table S1**. Study characteristics. **Table S2**. Risk of bias table by study. **Table S3**. Microscopy methodology used in included studies. **Table S4**. Factors associated with WBC count at baseline: univariable regression models, showing % change in WBC count. **Table S5**. Relative changes in mean WBC over time: univariable analyses showing mean % change compared to baseline WBC. **Table S6**. Distribution of different WBC types over time and by age group. **Table S7**. Change in estimated parasite density when assumed WBC used, by age group. **Table S8**. Number of falciparum malaria patients for whom the hyperparasitemic status was misclassified using assumed WBC count for estimation of parasite density. **Table S9**. Additive inflation factor for the slope of parasite clearance when the same assumed same value of WBC count was used in parasite estimation on day 0 and day 2. **Table S10**. Error in estimation of parasite clearance half-life PC_1/2_ when the same assumed value of WBC count was used in parasite estimation on day 0 and day 2.

**Additional file 2: Fig. S1**. Geometric mean baseline WBC count in children aged <1 year infected with P. falciparum, by study. **Fig. S2**. Geometric mean baseline WBC count in children aged 1-4 years infected with P. falciparum, by study. **Fig. S3**. Geometric mean baseline WBC count in children aged 5-14 years infected with P. falciparum, by study. **Fig. S4**. Geometric mean baseline WBC count in adults aged 15+ years infected with P. falciparum, by study. **Fig. S5**. Geometric mean baseline WBC count in children aged <5 years infected with P. vivax, by study. **Fig. S6**. Geometric mean baseline WBC count in children aged 5-14 years infected with P. vivax, by study. **Fig. S7**. Geometric mean baseline WBC count in adults aged 15+ years infected with P. vivax, by study.

## Data Availability

The data that support the findings of this study are available for access via the WorldWide Antimalarial Resistance Network (WWARN.org). Requests for access will be reviewed by a Data Access Committee to ensure that use of data protects the interests of the participants and researchers according to the terms of ethics approval and principles of equitable data sharing. Requests can be submitted by email to malariaDAC@iddo.org via the Data Access Form available at https://www.wwarn.org/working-together/sharing-accessing-data/accessing-data. The WWARN platform is registered with the Registry of Research Data Repositories (re3data.org).

## Estimation of inflation factors for estimation of parasite density and parasite clearance

1. *Estimation of a single parasite density*

Parasite density per µL, P(WBC), when parasite are counted per thick smear, can be calculated from the following formula:

P (WBC) = Number of parasites counted × total WBC count per µL ÷ number of WBCs counted

If Pm = P(measured WBC) is the parasite density estimated using measured WBC count, and

Pa = P(assumed WBC) is the parasite density estimated using an assume value of WBC count, then

$$\text{Pa/Pm}=\text{assumed WBC/measured WBC}$$

$$\text{Pa}\,=\,\text{Pm}\times\text{assumed WBC/measured WBC}$$

The inflation factor when using the assumed WBC count instead of the measured WBC count is equal to INF = assumed WBC/measured WBC.

2. *Estimation of parasite clearance from parasitaemia measurements on day 0 and 2*

Slope b of the regression line log(P_t_) = a – b × t, where P_t_ is the parasitaemia measurement at time t, represents the fraction by which parasite count falls per unit time. Parasite clearance half-life PC_1/2_ defined as log(2)/b is the time needed for parasitaemia to reduce by half.

Based on two parasite density measurements estimated using measured WBC count Pm_0_ on day 0 and Pm_2_ on day 2, slope of parasite clearance curve can be calculated as:

Slope_t_ = (log(Pm_0_) – log(Pm_2_))/48 = log(Pm_0_/Pm_2_)/48.

When using parasitaemia count based on the assumed value of WBC count, the slope can be expressed as:

Slope_a_ = log((Pm _0_× INF_0_)/(Pm_2_ × INF_2_))/48 = log(Pm_0_/Pm_2_)/48 + log(INF_0_/INF_2_)/48 = 

Slope_t_ + log(INF_0_/INF_2_)/48 = Slope _t_+ log(measured WBC_2_/measured WBC_0_)/48

where INF_*i*_ is an inflation factor on day *i*, and WBC_i_ is WBC measured on day *i*.

Therefore there is an additive inflation factor associated with estimation of slope of parasite clearance using assumed value of WBC count, which is equal to

INF_S_ = log(measured WBC_2_/measured WBC_0_)/48.

Consequently, parasite clearance half-life PC_1/2_ estimated using the assumed value of WBC count is equal to:

PCE_1/2a_ = log(2)/Slope_a_ = log(2)/(Slope_t_ + INF_S_).

## Abbreviations

Hb: Haemoglobin
IPD: Individual patient data
IQR: Interquartile range
N/A: Not applicable
PC_1/2_: Parasite clearance half-life
RBC: Red blood cell
RCT: Randomized controlled trial
WAZ: Weight-for-age Z-score
WBC: White blood cell
WHO: World Health Organization
WHZ: Weight-for-height Z-score
WWARN: WorldWide Antimalarial Resistance Network

## References

[1] WHO (2023). World malaria report 2022.

[2] Dini S, Douglas NM, Poespoprodjo JR, Kenangalem E, Sugiarto P, Plumb ID (2020). The risk of morbidity and mortality following recurrent malaria in Papua, Indonesia: a retrospective cohort study. BMC Med.

[3] Akinosoglou KS, Solomou EE, Gogos CA (2012). Malaria: a haematological disease. Hematol Amst Neth.

[4] Bakhubaira S (2013). Hematological parameters in severe complicated *Plasmodium falciparum* malaria among adults in Aden. Turk J Haematol.

[5] Squire DS, Asmah RH, Brown CA, Adjei DN, Obeng-Nkrumah N, Ayeh-Kumi PF (2016). Effect of *Plasmodium falciparum* malaria parasites on haematological parameters in Ghanaian children. J Parasit Dis.

[6] Wickramasinghe SN, Abdalla SH (2000). Blood and bone marrow changes in malaria. Baillieres Best Pract Res Clin Haematol.

[7] McKenzie FE, Prudhomme WA, Magill AJ, Forney JR, Permpanich B, Lucas C (2005). White blood cell counts and malaria. J Infect Dis.

[8] Tang H, Jing J, Bo D, Xu D (2012). Biological variations of leukocyte numerical and morphologic parameters determined by UniCel DxH 800 hematology analyzer. Arch Pathol Lab Med.

[9] Jadhav UM (2003). Prognostic implications of white cell differential count and white cell morphology in malaria. J Postgrad Med.

[10] Tobón-Castaño A, Mesa-Echeverry E, Miranda-Arboleda AF (2015). Leukogram profile and clinical status in vivax and falciparum malaria patients from Colombia. J Trop Med.

[11] Kimbi HK, Sumbele IUN, Nweboh M, Anchang-Kimbi JK, Lum E, Nana Y (2013). Malaria and haematologic parameters of pupils at different altitudes along the slope of Mount Cameroon: a cross-sectional study. Malar J.

[12] Eriksson B, Hellgren U, Rombo L (1989). Changes in erythrocyte sedimentation rate, C-reactive protein and hematological parameters in patients with acute malaria. Scand J Infect Dis.

[13] Erhart LM, Yingyuen K, Chuanak N, Buathong N, Laoboonchai A, Miller RS (2004). Hematologic and clinical indices of malaria in a semi-immune population of western Thailand. Am J Trop Med Hyg.

[14] Maghendji-Nzondo S, Nzoughe H, Lemamy GJ, Kouna LC, Pegha-Moukandja I, Lekoulou F (2016). Prevalence of malaria, prevention measures, and main clinical features in febrile children admitted to the Franceville Regional Hospital. Gabon Parasite.

[15] Abdalla S, Pasvol G (2004). Malaria: a hematological perspective.

[16] Olliaro P, Djimdé A, Karema C, Mårtensson A, Ndiaye JL, Sirima SB (2011). Standardised versus actual white cell counts in estimating thick film parasitaemia in African children under five. Trop Med Int Health.

[17] Diakite M, Miura K, Diouf A, Konate D, Keita AS, Doumbia S (2016). Hematological indices in malian children change significantly during a malaria season and with increasing age: implications for malaria epidemiological studies. Am J Trop Med Hyg.

[18] Tangpukdee N, Yew HS, Krudsood S, Punyapradit N, Somwong W, Looareesuwan S (2008). Dynamic changes in white blood cell counts in uncomplicated *Plasmodium falciparum* and P. vivax malaria. Parasitol Int.

[19] Dhorda M, Ba EH, Baird KJ, Barnwell J, Bell D, Carter JY (2020). Towards harmonization of microscopy methods for malaria clinical research studies. Malar J.

[20] WHO (2015). Microscopy for the detection, identification and quantification of malaria parasites on stained thick and thin blood films in research settings (version 1.0): procedure: methods manual.

[21] Hammami I, Nuel G, Garcia A (2013). Statistical properties of parasite density estimators in malaria. PLoS ONE.

[22] Greenwood BM, Armstrong JR (1991). Comparison of two simple methods for determining malaria parasite density. Trans R Soc Trop Med Hyg.

[23] Mischlinger J, Pitzinger P, Veletzky L, Groger M, Zoleko-Manego R, Adegnika AA (2018). Validity and reliability of methods to microscopically detect and quantify malaria parasitaemia. Trop Med Int Health.

[24] Bowers KM, Bell D, Chiodini PL, Barnwell J, Incardona S, Yen S (2009). Inter-rater reliability of malaria parasite counts and comparison of methods. Malar J.

[25] Das D, Dahal P, Dhorda M, Citarella BW, Kennon K, Stepniewska K (2021). A systematic literature review of microscopy methods reported in malaria clinical trials. Am J Trop Med Hyg.

[26] Adu-Gyasi D, Adams M, Amoako S, Mahama E, Nsoh M, Amenga-Etego S (2012). Estimating malaria parasite density: assumed white blood cell count of 10,000/μl of blood is appropriate measure in Central Ghana. Malar J.

[27] Liu H, Feng G, Zeng W, Li X, Bai Y, Deng S (2016). A more appropriate white blood cell count for estimating malaria parasite density in Plasmodium vivax patients in northeastern Myanmar.. Acta Trop.

[28] Rishikesh K, Madivala SK, Prabhu P, Kamath A, Ashok H, Vidyasagar S (2015). Surmised total leucocyte counts miscalculate the parasite index of *Plasmodium vivax* malaria patients of tertiary and primary care settings in South-Western India. Malar J.

[29] Omalu IC, Oguche S, Gyang VP, Akindigh TM, Egah DZ, Gokop B (2008). Standard white blood cell count for malaria density estimation: a need for review?. Ann Trop Med Public Health.

[30] Jeremiah ZA, Uko EK (2007). Comparative analysis of malaria parasite density using actual and assumed white blood cell counts. Ann Trop Paediatr.

[31] Bilal JA, Gasim GI, Karsani AH, Elbashir LM, Adam I (2016). Malaria parasite density estimation using actual and assumed white blood cells count in children in Eastern Sudan. J Trop Pediatr.

[32] Alves-Junior ER, Gomes LT, Ribatski-Silva D, Mendes CRJ, Leal-Santos FA, Simões LR (2014). Assumed white blood cell count of 8000 cells/μL overestimates malaria parasite density in the Brazilian Amazon. PLoS ONE.

[33] Haggaz AD, Elbashir LM, Adam GK, Rayis DA, Adam I (2014). Estimating malaria parasite density among pregnant women at central Sudan using actual and assumed white blood cell count. Malar J.

[34] Laman M, Moore BR, Benjamin J, Padapu N, Tarongka N, Siba P (2014). Comparison of an assumed versus measured leucocyte count in parasite density calculations in Papua New Guinean children with uncomplicated malaria. Malar J.

[35] WWARN Parasite Clearance Study Group (2015). Baseline data of parasite clearance in patients with falciparum malaria treated with an artemisinin derivative: an individual patient data meta-analysis. Malar J.

[36] Murthy GL, Sahay RK, Srinivasan VR, Upadhaya AC, Shantaram V, Gayatri K (2000). Clinical profile of falciparum malaria in a tertiary care hospital. J Indian Med Assoc.

[37] WHO (2012). Management of severe and complicated malaria: a practical handbook.

[38] Clinical Data Management and Analysis Plan. Worldwide Antimalarial Resistance Network. https://www.wwarn.org/tools-resources/clinical-data-management-and-analysis-plan Accessed from 12 Apr 2019.

[39] WWARN Falciparum Haematology Study Group (2022). Haematological consequences of acute uncomplicated falciparum malaria: a WorldWide antimalarial resistance network pooled analysis of individual patient data. BMC Med.

[40] Commons RJ, Simpson JA, Thriemer K, Chu CS, Douglas NM, Abreha T (2019). The haematological consequences of *Plasmodium vivax* malaria after chloroquine treatment with and without primaquine: a WorldWide antimalarial resistance network systematic review and individual patient data meta-analysis. BMC Med.

[41] WWARN. Data Access Committee (DAC). Worldwide Antimalarial Resistance Network. 2019. https://www.wwarn.org/working-together/sharing-accessing-data/data-access-committee-dac Accessed from 14 Sep 2019.

[42] WWARN. Terms of Submission. Worldwide Antimalarial Resistance Network. 2015 https://www.wwarn.org/tools-resources/terms-submission Accessed from 14 Sep 2019.

[43] Bhatt S, Weiss DJ, Cameron E, Bisanzio D, Mappin B, Dalrymple U (2015). The effect of malaria control on *Plasmodium falciparum* in Africa between 2000 and 2015. Nature.

[44] Commons RJ, Simpson JA, Thriemer K, Hossain MS, Douglas NM, Humphreys GS (2019). Risk of *Plasmodium vivax* parasitaemia after *Plasmodium falciparum* infection: a systematic review and meta-analysis. Lancet Infect Dis.

[45] Haematology Normal Adult Reference Ranges. https://www.royalwolverhampton.nhs.uk/services/service-directory-a-z/pathology-services/departments/haematology/haematology-normal-adult-reference-ranges/ Accessed from 29 Apr 2019.

[46] Full Blood Count. North Bristol NHS Trust. https://www.nbt.nhs.uk/severn-pathology/requesting/test-information/full-blood-count Accessed from 29 Apr 2019.

[47] DAIDS Adverse Event Grading Tables | DAIDS Regulatory Support Center (RSC). https://rsc.niaid.nih.gov/clinical-research-sites/daids-adverse-event-grading-tables Accessed from 14 Aug 2019.

[48] Lee SJ, Stepniewska K, Anstey N, Ashley E, Barnes K, Binh TQ (2008). The relationship between the haemoglobin concentration and the haematocrit in *Plasmodium falciparum* malaria. Malar J.

[49] WHO (2019). Anthro (version 3.2.2, January 2011) and macros.

[50] WHO (2010). Guidelines for the Treatment of Malaria.

[51] WHO (2015). Guidelines for the treatment of malaria.

[52] Collet, D. Modelling Survival Data in Medical Research—CRC Press Book. https://www.crcpress.com/Modelling-Survival-Data-in-Medical-Research/Collett/p/book/9781439856789 Accessed from 7 May 2019.

[53] Flegg JA, Guerin PJ, White NJ, Stepniewska K (2011). Standardizing the measurement of parasite clearance in falciparum malaria: the parasite clearance estimator. Malar J.

[54] WHO (2018). Artemisinin resistance and artemisinin-based combination therapy efficacy: status report.

[55] WWARN K13 Genotype-Phenotype Study Group (2019). Association of mutations in the *Plasmodium falciparum* Kelch13 gene (Pf3D7_1343700) with parasite clearance rates after artemisinin-based treatments-a WWARN individual patient data meta-analysis. BMC Med.

[56] Modiano D, Sirima BS, Konaté A, Sanou I, Sawadogo A (2001). Leucocytosis in severe malaria. Trans R Soc Trop Med Hyg.

[57] Church J, Maitland K (2014). Invasive bacterial co-infection in African children with *Plasmodium falciparum* malaria: a systematic review. BMC Med.

[58] Naeim F, Nagesh Rao P, Song SX, Phan RT, Nagesh Rao P, Song SX, Phan RT (2018). Granulocytic Disorders. Naeim F Atlas of Hematopathology.

[59] Opata MM, Ibitokou SA, Carpio VH, Marshall KM, Dillon BE, Carl JC (2018). Protection by and maintenance of CD4 effector memory and effector T cell subsets in persistent malaria infection. PLoS Pathog.

[60] Kurup SP, Butler NS, Harty JT (2019). T cell-mediated immunity to malaria. Nat Rev Immunol.

[61] Obaldia N, Meibalan E, Sa JM, Ma S, Clark MA, Mejia P (2018). Bone marrow is a major parasite reservoir in *Plasmodium vivax* infection. MBio.

[62] Kho S, Qotrunnada L, Leonardo L, Andries B, Wardani PAI, Fricot A (2021). Hidden biomass of intact malaria parasites in the human spleen. N Engl J Med.

[63] WHO (2009). Methods for surveillance of antimalarial drug efficacy.

[64] Khoury DS, Zaloumis SG, Grigg MJ, Haque A, Davenport MP (2020). Malaria parasite clearance: what are we really measuring?. Trends Parasitol.

[65] White NJ (2017). Malaria parasite clearance. Malar J.

[66] Haddy TB, Rana SR, Castro O (1999). Benign ethnic neutropenia: what is a normal absolute neutrophil count?. J Lab Clin Med.

