# Supplementary material for: Variability in white blood cell count during uncomplicated malaria and implications for parasite density estimation: a WorldWide Antimalarial Resistance Network individual patient data meta-analysis
Source: Malar J. 2023 Jun 6;22:174. doi: 10.1186/s12936-023-04583-6 (PMC10243075; doi:10.1186/s12936-023-04583-6)
Supplement: Supplementary file 2 — Additional file 2: Fig. S1. Geometric mean baseline WBC count in children aged <1 year infected with P. falciparum, by study. Fig. S2. Geometric mean baseline WBC count in children aged 1-4 years infected with P. falciparum, by study. Fig. S3. Geometric mean baseline WBC count in children aged 5-14 years infected with P. falciparum, by study. Fig. S4. Geometric mean baseline WBC count in adults aged 15+ years infected with P. falciparum, by study. Fig. S5. Geometric mean baseline WBC count in children aged <5 years infected with P. vivax, by study. Fig. S6. Geometric mean baseline WBC count in children aged 5-14 years infected with P. vivax, by study. Fig. S7. Geometric mean baseline WBC count in adults aged 15+ years infected with P. vivax, by study. [file 12936_2023_4583_MOESM2_ESM.pdf]

## Additional figures

### *P. falciparum*

Additional file 1: Figure. S1 Geometric mean baseline WBC count in children aged <1 year infected with *P. falciparum*, by study

Additional file 1: Figure. S2 Geometric mean baseline WBC count in children aged 1-4 years infected with *P. falciparum*, by study

Additional file 1: Figure. S3 Geometric mean baseline WBC count in children aged 5-14 years infected with *P. falciparum*, by study

Additional file 1: Figure. S4 Geometric mean baseline WBC count in adults aged 15+ years infected with *P. falciparum*, by study

### *P. vivax*

Additional file 1: Figure. S5 Geometric mean baseline WBC count in children aged <5 years infected with *P. vivax*, by study

Additional file 1: Figure. S6 Geometric mean baseline WBC count in children aged 5-14 years infected with *P. vivax*, by study

Additional file 1: Figure. S7 Geometric mean baseline WBC count in adults aged 15+ years infected with *P. vivax*, by study

Additional file 1: Fig. S1. Geometric mean baseline WBC count in children aged <1 year infected with *P. falciparum*, by study ($I^2$ = 99.1%, p<0.001)

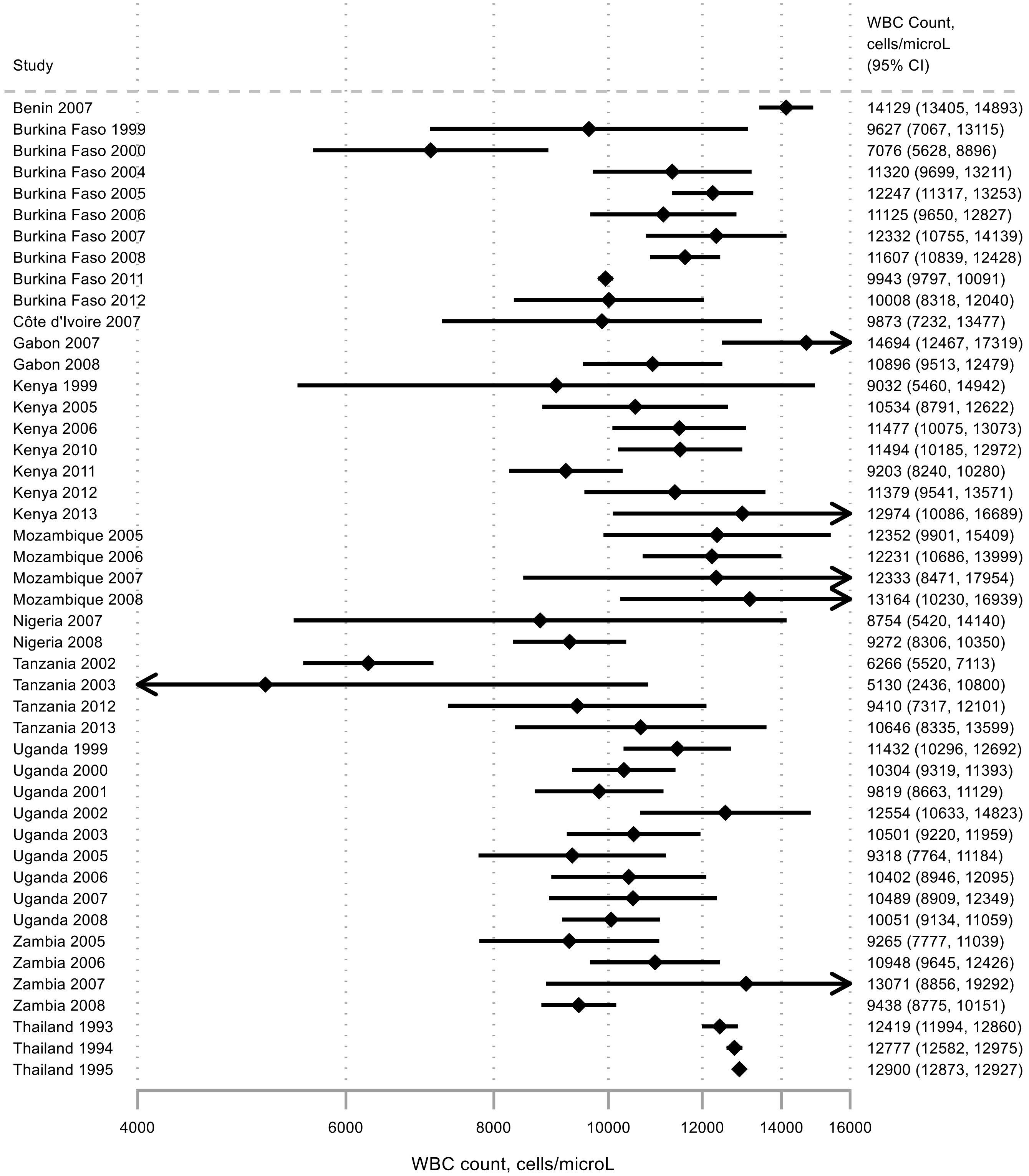


Study denotes country and first year of data collection. WBC count presented is the geometric mean baseline WBC count.

Additional file 1: Fig. S2. Geometric mean baseline WBC count in children aged 1-4 years infected with *P. falciparum*, by study ($I^2$ = 99.9%, p<0.001)

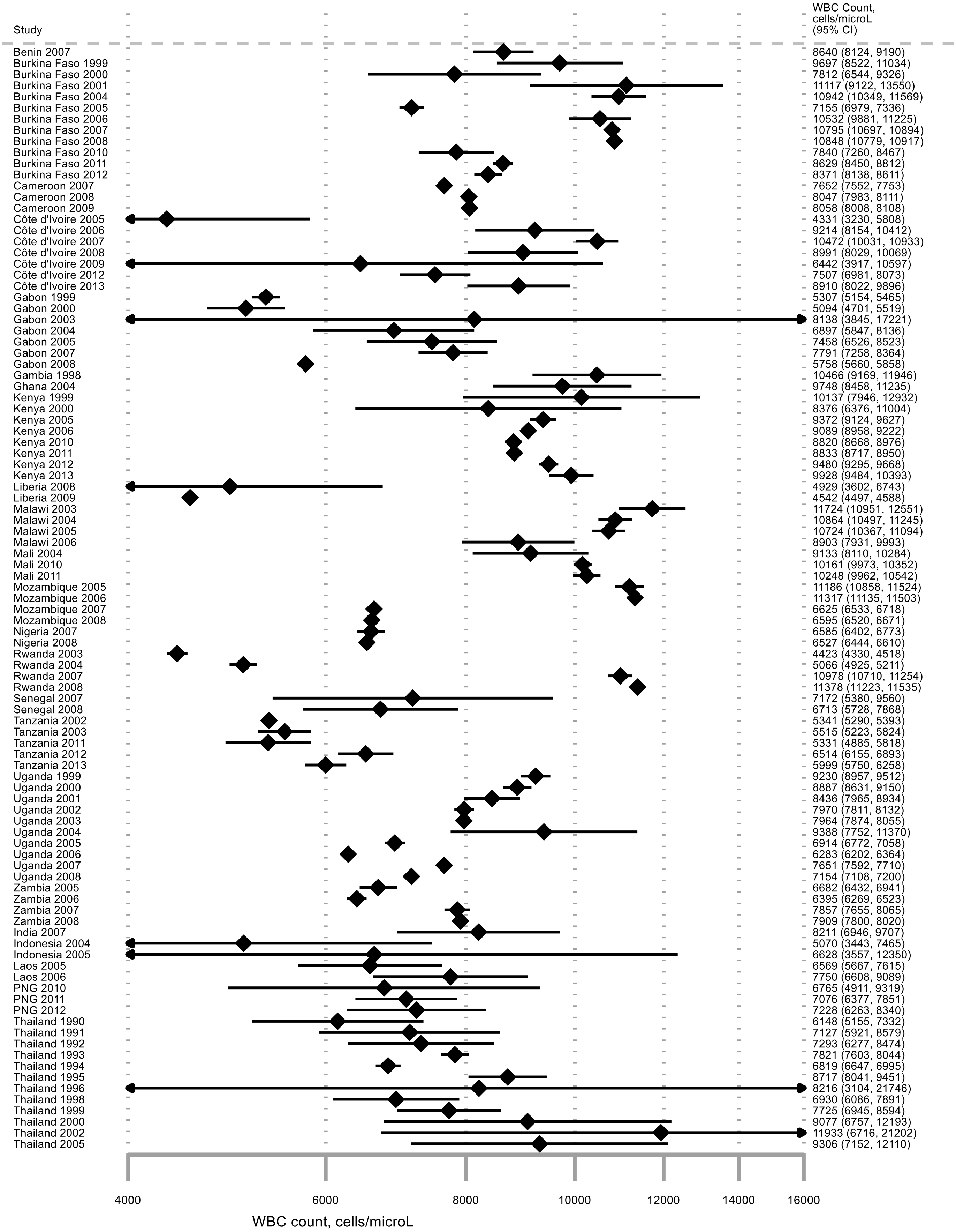


PNG = Papua New Guinea.

Study denotes country and first year of data collection. WBC count presented is the geometric mean baseline WBC count.

Additional file 1: Fig. S3.Geometric mean baseline WBC count in children aged 5-14 years infected with *P. falciparum*, by study ($I^2$ = 99.8%, p<0.001)

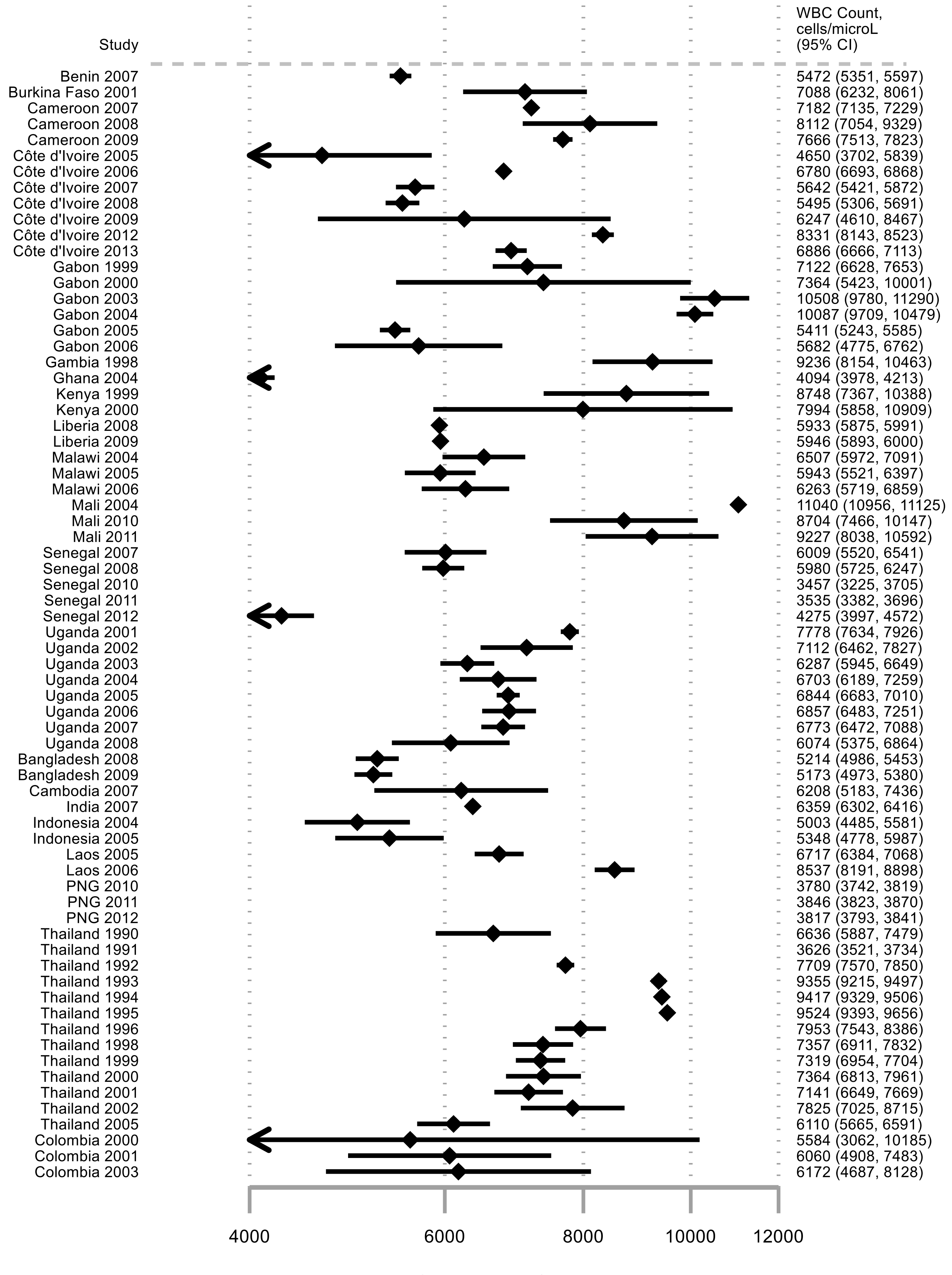


PNG = Papua New Guinea.

Study denotes country and first year of data collection. WBC count presented is the geometric mean baseline WBC count.

Additional file 1: Fig. S3. Geometric mean baseline WBC count in adults aged 15+ years infected with *P. falciparum*, by study ($I^2$ = 99.5%, p<0.001)

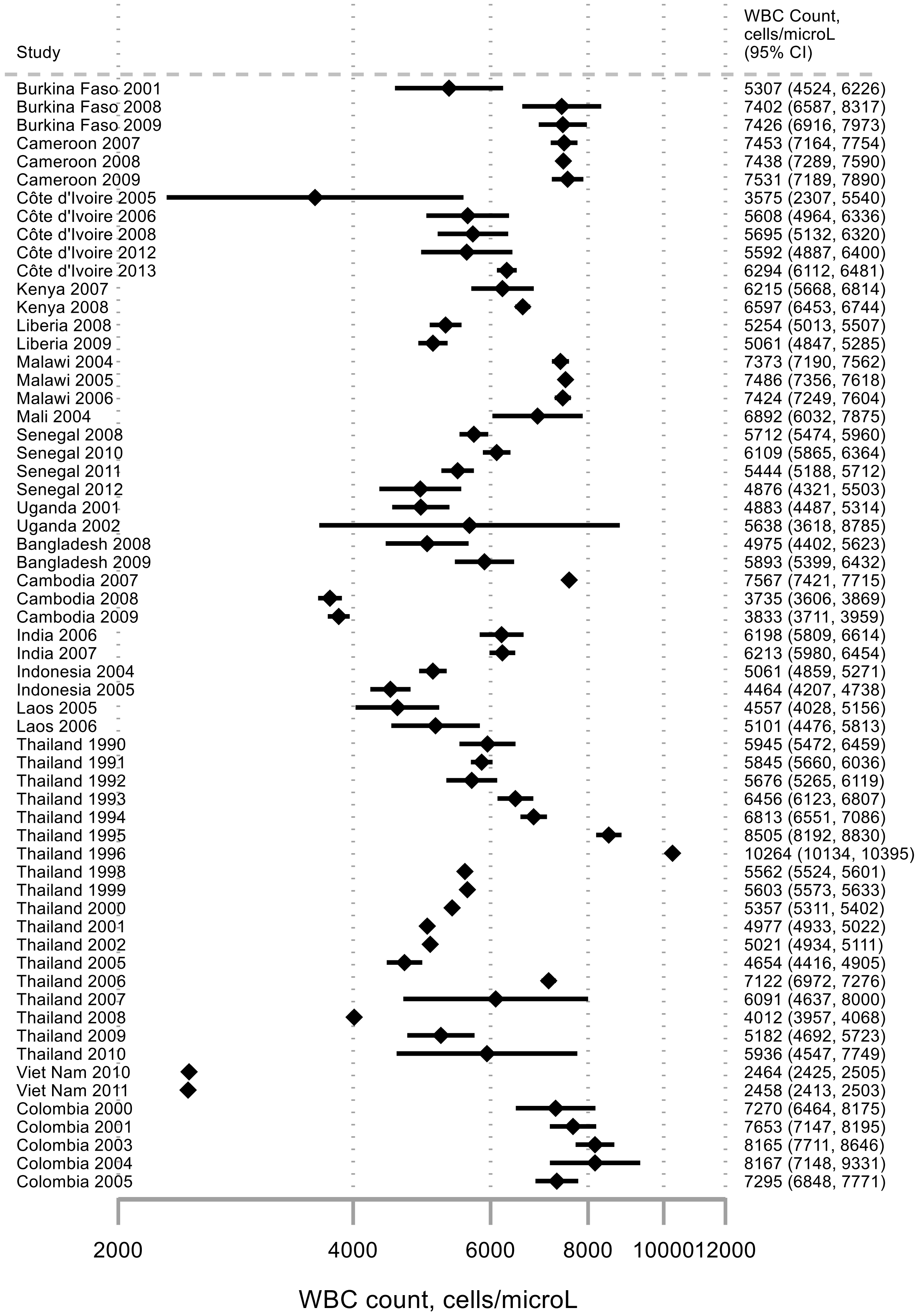


Study denotes country and first year of data collection. WBC count presented is the geometric mean baseline WBC count.

Additional file 1: Fig. S5.Geometric mean baseline WBC count in children aged <5 years infected with *P. vivax*, by study ($I^2$ = 83.1%, p<0.001)

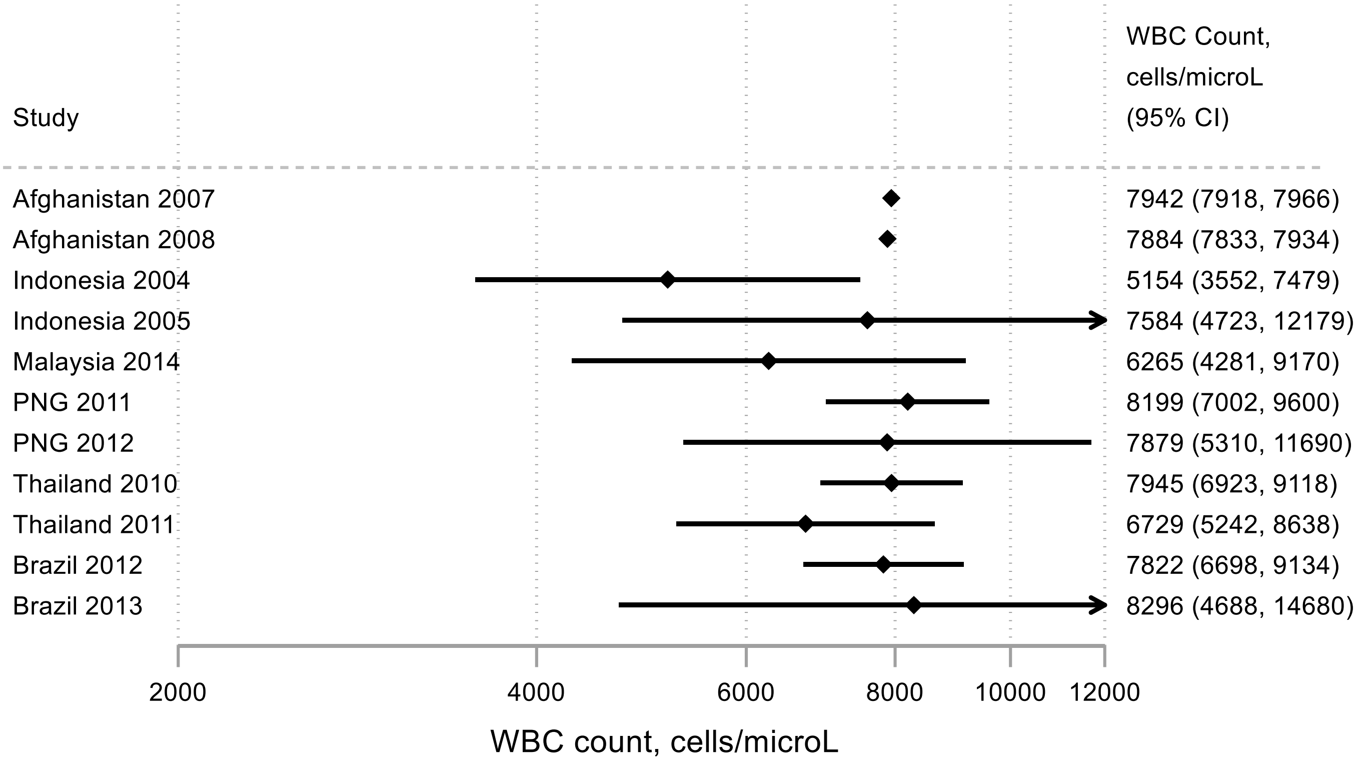


PNG = Papua New Guinea.

Study denotes country and first year of data collection. WBC count presented is the geometric mean baseline WBC count.

Additional file 1: Fig. S6.Geometric mean baseline WBC count in children aged 5-14 years infected with *P. vivax*, by study ($I^2$ = 97.5%, p<0.001)

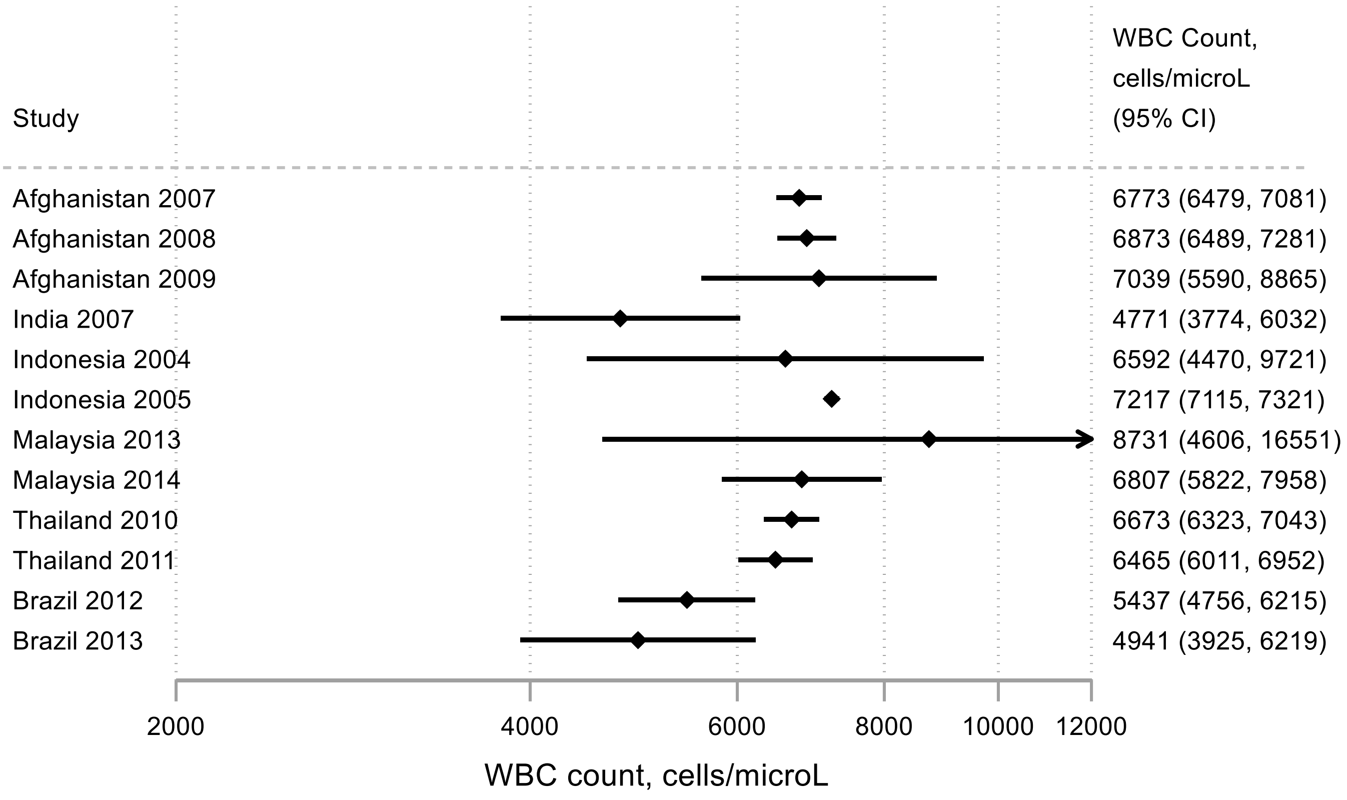


Study denotes country and first year of data collection. WBC count presented is the geometric mean baseline WBC count.

Additional file 1: Fig. S7.Geometric mean baseline WBC count in adults aged 15+ years infected with *P. vivax*, by study ($I^2$ = 99.1%, p<0.001)

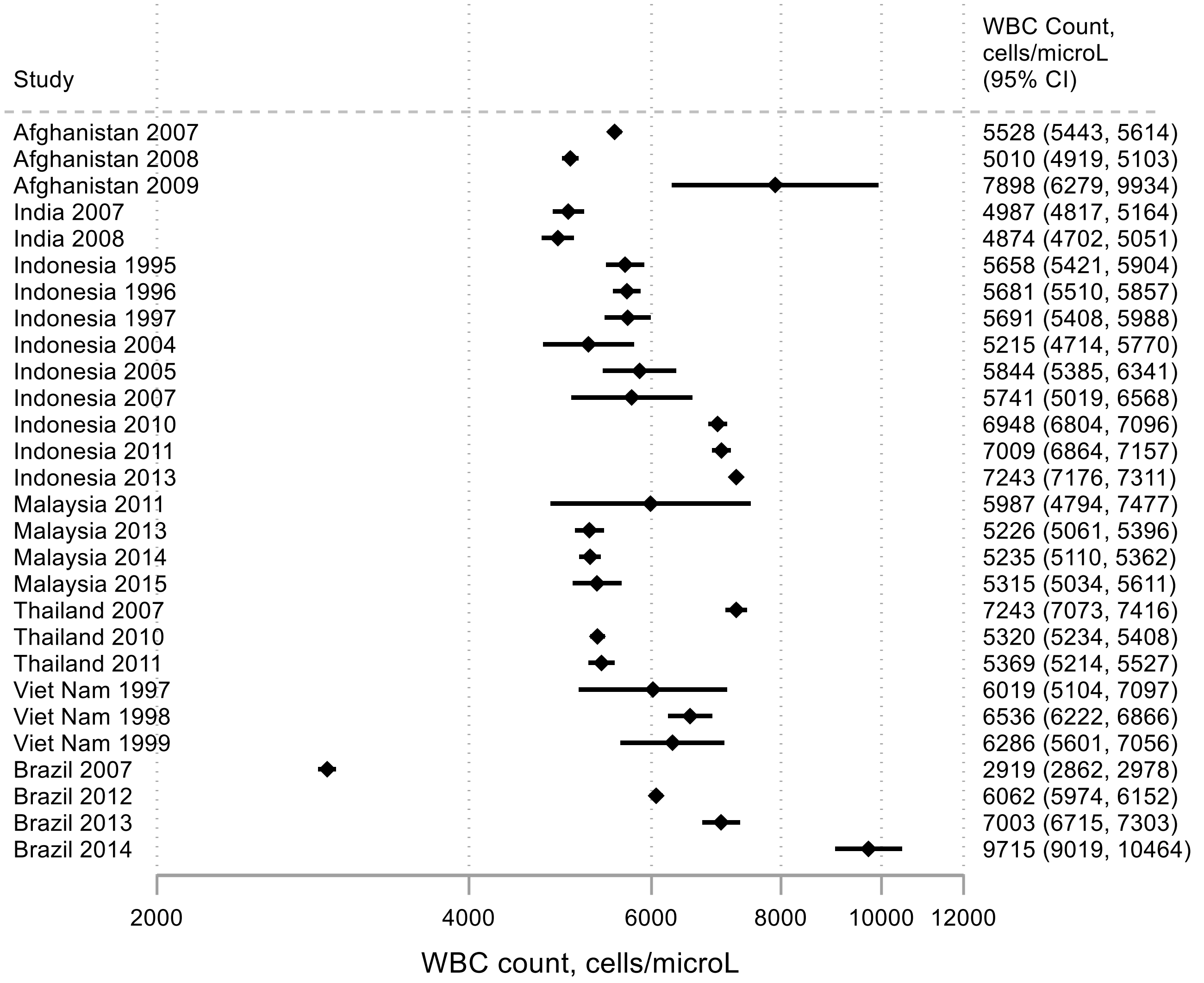


Study denotes country and first year of data collection. WBC count presented is the geometric mean baseline WBC count.
